# Estrogen receptors, ERK_1/2_ phosphorylation and reactive oxidizing species in red blood cells from patients with rheumatoid arthritis

**DOI:** 10.3389/fphys.2022.1061319

**Published:** 2022-12-05

**Authors:** Manuela Di Franco, Rosa Vona, Lucrezia Gambardella, Camilla Cittadini, Martina Favretti, Chiara Gioia, Elisabetta Straface, Donatella Pietraforte

**Affiliations:** ^1^ Rheumatology Unit, Department of Clinical Internal, Anesthetic and Cardiovascular Sciences, Sapienza University of Rome, Rome, Italy; ^2^ Biomarkers Unit, Center for Gender-Specific Medicine, National Institute of Health (ISS), Rome, Italy; ^3^ Core Facilities, National Institute of Health (ISS), Rome, Italy

**Keywords:** rheumatoid arthritis, inflammation, oxidative stress, red blood cells, estrogen receptors

## Abstract

Red blood cells (RBCs) are recognized to be important pathogenetic determinants in several human cardiovascular diseases (CVD). Undergoing to functional alterations when submitted to risk factors, RBCs modify their own intracellular signaling and the redox balance, shift their status from antioxidant defense to pro-oxidant agents, become a potent atherogenic stimulus playing a key role in the dysregulation of the vascular homeostasis favoring the developing and progression of CVD. Rheumatoid arthritis (RA) is a chronic autoimmune disease associated with a significantly increased risk of cardiovascular mortality with a prevalence from two to five more likely in woman, mainly attributed to accelerated atherosclerosis. The purpose of this study was to correlate the RA disease activity and the RBCs functional characteristics. Thirty-two women (aged more than 18 years) with RA, and 25 age-matched healthy women were included in this study. The disease activity, measured as the number of swollen and painful joints (DAS-28), was correlated with 1) the expression of RBCs estrogen receptors, which modulate the RBC intracellular signaling, 2) the activation of the estrogen-linked kinase ERK_½_, which is a key regulator of RBC adhesion and survival, and 3) the levels of inflammatory- and oxidative stress-related biomarkers, such as the acute-phase reactants, the antioxidant capacity of plasma, the reactive oxidizing species formation and 3-nitrotyrosine. All the biomarkers were evaluated in RA patients at baseline and 6 months after treatment with disease-modifying anti-rheumatic drugs (DMARDs). We found, for the first times, that in RA patients 1) the DAS-28 correlated with RBC ER-α expression, and did not correlate with total antioxidant capacity of plasma; 2) the RBC ER-α expression correlated with systemic inflammatory biomarkers and oxidative stress parameters, as well as ERK_½_ phosphorylation; and 3) the DMARDs treatments improved the clinical condition measured by DAS-28 score decrease, although the RBCs appeared to be more prone to pro-oxidant status associated to the expression of survival molecules. These findings represent an important advance in the study of RA determinants favoring the developing of CVD, because strongly suggest that RBCs could also participate in the vascular homeostasis through fine modulation of an intracellular signal linked to the ER-α.

## Introduction

Besides their role as oxygen/carbon dioxide carriers, red blood cells (RBCs) play a key role in vascular homeostasis under physiological conditions, having a fundamental role in redox regulation and intracellular signaling (Mahdi et al., 2021). RBCs are indeed committed to maintain the suitable balance between the blood antioxidant and pro-oxidant status, as well as to the control of the vasodilatation through the transport and release of nitric oxide (NO) (Mahdi et al., 2021). They are excellent scavengers of reactive oxygen (ROS) and nitrogen (RNS) species. In fact, being equipped with an efficient antioxidant system, they control the oxidizing species that, in inflammatory conditions, are generated in the vascular system by enzymes such as nicotinamide adenine dinucleotide phosphate (NADPH) oxidase, xanthine oxidoreductase and endothelial NO synthase (eNOS). In addition, RBCs control the yields of the ROS and RNS generated endogenously by partially oxygenated hemoglobin, such as the superoxide anion (O_2_^•^), or the NO generated by deoxyhemoglobin (Da Fonseca et al., 2019). Furthermore, as recently reported, they control the oxidizing species generated by an active and functional eNOS isoform localized in the plasma membrane and in the cytoplasm (Kundu et al., 2012; Veselinovic et al., 2014; Ferreira et al., 2021).

The capable RBCs antioxidant machinery includes both non-enzymatic low molecular weight antioxidants (glutathione, ascorbic acid, and vitamin E) and enzymatic antioxidants (superoxide dismutase, catalase, glutathione peroxidase, peroxiredoxin, glutathione peroxidase and the thioredoxin reductase/thioredoxin system) all being committed to directly scavenge the produced oxidant species or protect the critical thiols in key enzymes/proteins, maintaining the suitable intracellular redox state and signalling.

Importantly, RBCs are by now recognized as real-time biomarkers and pathogenetic determinants in several human cardiovascular diseases (CVD) (Smolen et al., 2018; Rezuș et al., 2021; Mahdi et al., 2021). A change in their morphological and functional characteristics (adhesivity, aggregability, and deformability) has been detected in several human CVD-linked pathologic conditions, mostly in those displaying systemic oxidative stress as a hallmark. For instance, changes in RBC adhesiveness/aggregation and morphology have been detected in inflammatory conditions, plaque instability and atheroma progression in patients with coronary artery disease (Quiñonez-Flores et al., 2016; Smolen et al., 2018; Rezuș et al., 2021; Mahdi et al., 2021). Redox changes of RBCs are believed to be a potent atherogenic stimulus contributing to the deposition of cholesterol at the atherosclerotic plaque (Smallwood et al., 2018) and playing a role in the pathogenesis of hypertension (García-Sánchez et al., 2020) and stroke (Mateen et al., 2016).

Under pathophysiological conditions, changes in the redox state of RBCs shift their role from antioxidant defense to pro-oxidant state (Mahdi et al., 2021). Oxidized RBCs undergo a rearrangement of cytoskeletal proteins, oxidation, and loss of lipid asymmetry. They become more rigid, undergo lysis, and release cytotoxic species in the vasculature that can modulate certain blood cell functions (Minetti et al., 2007) and induce dysfunction and progression of CVD (Mahdi et al., 2021).

Rheumatoid arthritis (RA) is a chronic autoimmune disease characterized by a strong systemic inflammatory condition that accelerates development of atherosclerosis caused by the inflammation-mediated endothelial dysfunction and vascular damage in the blood vessels. Compared to men, women are 2–5 times more likely to develop RA. They have a high prevalence of CVD, hypercoagulability, and consequently increased risk of developing cardiovascular events such as stroke and myocardial infarction (Quiñonez-Flores et al., 2016; Smallwood et al., 2018; Smolen et al., 2018; Rezuș et al., 2021).

A common feature of autoimmune inflammatory diseases is the increased formation of ROS, RNS, and chloride-derived species produced by activated immune cells in inflamed tissues (Smallwood et al., 2018; García-Sánchez et al., 2020; Stamp et al., 2012). Oxidative stress has also been recognized contributing to RA pathogenesis due to the inflammatory conditions characterizing this disease (Mateen et al., 2016; Quiñonez-Flores et al., 2016; Smallwood et al., 2018; Da Fonseca et al., 2019; Ferreira et al., 2021). The infiltration of immune cells into the synovial joint lining, the release of the pro-inflammatory cytokines, the redox imbalance linked to the release of oxidizing species by activated cells resulting in impaired intracellular signaling, as well as the impairment of the endogenous antioxidant system, are responsible for the oxidative damage and destruction of joints ultimately. All these conditions lead to substantial disability in RA patients (Kundu et al., 2012; Veselinovic et al., 2014; Quiñonez-Flores et al., 2016). In addition, a positively correlations between ROS and RNS, produced by the inflammatory conditions, the occurrence of rheumatic diseases and the accelerated joint destruction has been reported (Quiñonez-Flores et al., 2016). In RA patients, ROS, mainly O_2_^•^ and hydrogen peroxide (H_2_O_2_) and RNS (NO and its derived oxidants) are generated by chondrocytes in the inflamed joint, activated macrophages in the synovial membrane and by the activated neutrophils in the synovial cavity, leading to oxidative damage to cartilage, extracellular matrix, collagen, and proteoglycans (Mateen et al., 2016).

Furthermore, ROS and RNS are known to induce lipid peroxidation that yields to the formation of lipid hydroperoxides, and electrophilic reactive lipid species end products, including malondialdehyde (MDA), which can form adducts with proteins able to modify their activity and function (Ferreira et al., 2021).

Recently, our group demonstrated that RBCs express both estrogen receptors (ER-α and ER-β), that they are functionally active (Vona et al., 2019) and involved in the phosphorylation of some key kinases, such as ERK_1/2_, AKT/PI3K and P38, which regulate cell adhesion and survival (Roskoski, 2012). In addition, as for endothelial cells, RBCs produce NO by phosphorylation of intracellular isoform of eNOS, whose activity is modulated by ER-α and ER-β.

In a previous study, we found that systemic oxidative stress occurring in RA patients did not interfere significantly with the redox state of RBCs, with a mild reactive oxidizing species production and a mild reduction of total thiol content having been detected in RBCs from RA patients. However, we found that these cells showed a significant up-regulation of survival molecules, such as the activated phosphorylated form of the mitogen-activated protein kinase (MAPK) ERK_1/2_ (p-ERK_1/2_) and survivin. The phosphorylation of specific tyrosine and threonine residues of target proteins mediated by p-ERK_1/2_ regulates fundamental cellular activities, such as cell adhesion, survival, senescence, and aging (Di Franco et al., 2015; Zou et al., 2019). In several diseases, including cancer, survivin is the most studied protein involved in the inhibition of apoptotic protein. Instead, ERK_1/2_ activation, plays a key role in several cellular signaling pathways, cell proliferation and programmed cell death (Bernardo et al., 2020).

Based on these data and to shed light on the modulation of the RBCs intracellular signaling, linked to the ERs in RA disease, the aim of this work was to evaluate the likely correlation of ERs expression with the disease activity, the levels of ERK_1/2_ phosphorylation and the occurrence of the expression of biomarkers related to oxidative and inflammatory stress. These assessments were made before and after 6 months of treatment with appropriate disease-modifying anti-rheumatic drugs (DMARDs).

## Materials and methods

### Patients

Thirty-two women aged more than 18 years (19 early- and 13 long-standing patients) affected by RA diagnosed according to 2010 ACR (American College of Rheumatology) criteria, coming from Early Arthritis Clinic and 25 age-matched healthy women (HD), were recruited at the Rheumatology Unit of Sapienza University of Rome. The study was reviewed and approved by the Local Ethical Committee at the Sapienza University. A written informed consent was obtained from all patients. All clinical investigations have been conducted according to the principles expressed in the Declaration of laboratory data to measure the Disease Activity Score (DAS-28), which takes into account the number of swollen and painful joints (out of 28 joints), acute-phase reactants such as erythrocyte sedimentation rate (ESR) and C-reactive protein (CRP), and patient’s global health. Smokers and patients with hypercholesterolemia, arterial hypertension, cardiovascular diseases, type 1 and 2 diabetes and cancer were excluded from the study. In particular, two groups of RA patients were evaluated: early- and long-standing disease patients. The first group of patients was enrolled before the treatment, while the second had been treated with methotrexate and did not respond to therapy. All patients were assessed at baseline and after 6 months (follow up) of treatment with the DMARDs methotrexate and anti-TNF-α, respectively.

### Red blood cells isolation

Fresh human blood from healthy donors was drawn into heparinized tubes. For RBCs isolation, whole blood was centrifuged for 10 min at 1.500 g. The plasma and buffy coat were removed. RBCs were washed twice in isotonic PBS, pH 7.4, and suspended in the same buffer to the initial hematocrit concentration. No appreciable cell lysis was observed during the RBC preparation procedure.

### Red blood cells redox balance

To evaluate the formation of total intracellular reactive oxidizing species, RBCs (5 × 10^5^cells) were incubated in the Hanks’ balanced salt solution, pH 7.4, containing dihydrorhodamine 123 (DHR123; Molecular Probes). Samples were then analyzed with a fluorescence-activated cell-sorting (FACS) flow cytometer (Becton Dickinson, Mountain View, CA, United States). The median values of fluorescence intensity histograms were used to provide semi-quantitative evaluation of the oxidizing species production.

### Analytical cytology

RBCs were fixed with 3.7% formaldehyde in PBS (pH 7.4) for 10 min at room temperature, washed in the same buffer and permeabilized with 0.5% Triton X-100 in PBS for 5 min at room temperature. After washing with PBS, samples were incubated for 30 min at 37°C with monoclonal antibodies: anti-ER-α, anti-ER-β, anti-phosphorylated ERK_1/2_ (BD PharMingen, San Diego, CA), anti-survivin (Santa Cruz Biotechnology) and anti-3-nitrotyrosine (Sigma Aldrich). After, all samples were washed thrice in PBS to be then incubated with secondary antibody FITC-conjugated: anti-mouse (Invitrogen, Carlsbad, CA) or anti-rabbit (Invitrogen, Carlsbad, CA). All the samples were recorded with a FACScan flow cytometer (Becton-Dickinson, Mountain View, CA, United States) equipped with a 488 United Statesnm argon laser. At least 20, 000 events were acquired. The median values of fluorescence intensity histograms were used to provide a semi-quantitative analysis. Morphometric analyses were also employed to evaluate ER-α and ER-β distribution. All samples were mounted on glass cover slips with fluorescence mounting medium (Dako) and observed by intensified video microscopy (IVM) with an Olympus Microphot fluorescence microscope (Olympus Corporation, Tokyo, Japan) equipped with a Zeiss CCD camera.

### Evaluation of plasma total antioxidant capacity

To evaluate total antioxidant capacity (TAC) of plasma, a commercially colorimetric assay kit has been used following manufacturer’s instruction (Bio-Vision, Abcam Company).

### Statistical analyses

Cytofluorimetric results were statistically analyzed by using the nonparametric Kolmogorov–Smirnov test using Cell Quest Software. At least 20, 000 events were acquired. The median values of fluorescence intensity histograms were used to provide a semi-quantitative analysis. Student’s t-test was used for the statistical analysis of the collected data. Correlations were evaluated by using Pearson correlation (R correlation coefficient). *p* ≤ 0.05 values were considered statistically significant.

## Results

### Patient profile

In this study, two different groups of female RA patients comparable for age and showing different duration of the disease, early- and long-standing disease, have been investigated. As shown in Table 1, the disease duration was 5.5 times lower in early standing RA patients with respect to long-standing patients. When compared for the DAS-28 score, the early-standing patients showed high (71% of patients) and moderate (29% of patients) disease activity (DAS-28: 4.47 ± 1), while the long-standing patients showed low (35.7% of patients) and moderate (64.3% of patients) disease activity (DAS-28: 3.8 ± 0.8). The disease activity was sustained in early patients by the increased concentration of the acute-phase biomarkers, such as rheumatoid factor (RF), ESR and CRP, whose values were significantly higher (*p* ≤ 0.05) than in long-standing patients.

**TABLE 1:** Clinical characteristics of female RA patients.

Clinical characteristics	Early-standing disease (n = 19)	Long-standing disease (n = 13)	*p* Values
Age (y)	Median 52 (range 19–75)	Median 49 (range 19–77)	
Disease duration (weeks)	Median 21.5 weeks (range 3–52)	Median 118 weeks (range 54–324)	1.48
DAS 28 score	4.471 ± 1	3.8 ± 0.8	0.166
RF (IU/ml)	Median 183 (range 10—600)	Median 28 (range 8–137)	0.043*
ESR (mm/h)	Median 33.5 (range 4—76)	Median 14 (range 4—25)	0.012*
CRP (mg/L)	Median 12.88 (range 0.4—56)	Median 3.84 (range 0.1—15.8)	0.05*

RA, rheumatoid arthritis; DAS, 28 score, disease activity score; RF, rheumatoid factor; CRP, C reactive protein; ESR, erythrocyte sedimentation rate. *p ≤ 0.05 (Student’s t-test).

### Estrogen receptors expression and localization in RBCs from RA patients

**FIGURE 1:**
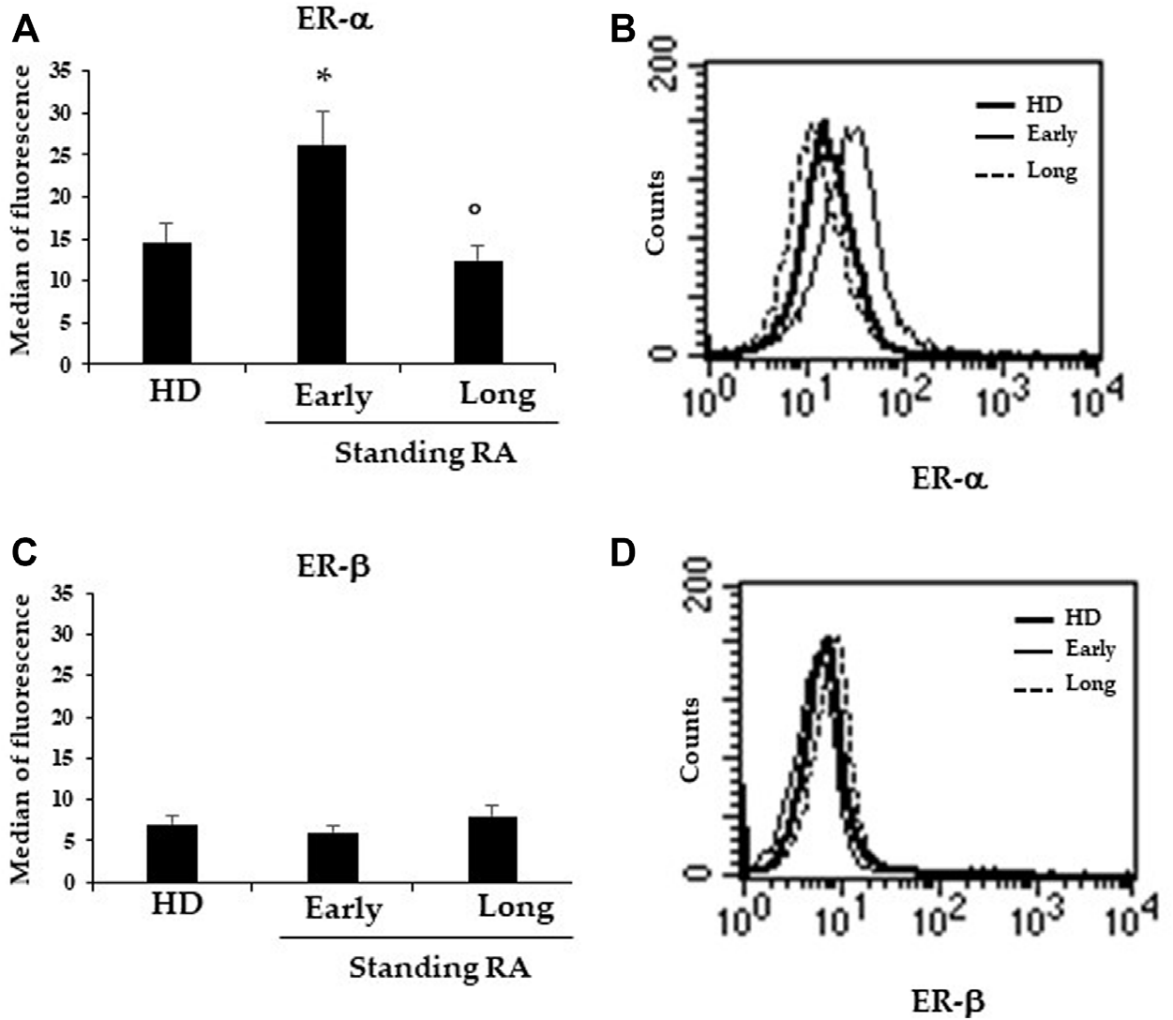
Expression of ERs in RBCs. **(A)** Flow cytometry analysis of ER-α expression in RBCs from 25 HD, 19 early- and 13 long-standing RA patients. **(B)** Dot plot showing the ER-β expression in RBCs from a representative HD, a representative early- and a representative long-standing RA patient. **(C)** ER-β expression in RBCs from 25 HD, 19 early- and 13 long-standing RA patients. **(D)** Dot plot showing ER-β expression in RBCs from a representative HD, a representative early- and a representative long-standing RA patient. *p ≤ 0.01.

**FIGURE 2:**
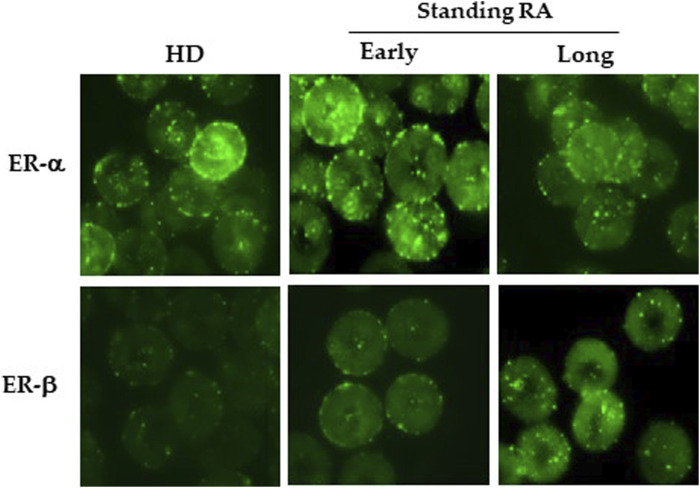
Localization of ERs in RBCs. Upper panels: Micrographs obtained by static cytometry showing ER-α distribution in RBCs from a representative HD, a representative early- and a representative long-standing RA patient. Lower panels: Micrographs obtained by static cytometry showing ER-β distribution in RBCs from a representative HD, a representative early- and a representative long-standing RA patient.

By using flow and static cytometry we therefore evaluated ER-α and ER-β expression (Figure 1) and localization (Figure 2) in RBCs from HD and early- and long-standing RA patients. As shown in Figure 1A, in comparison with HD, the semi-quantitative cytometric analysis showed that ER-α expression was increased significantly (*p* ≤ 0.01) in RBCs from early-patients while no significant differences were detected in RBCs from long-standing patients. Interestingly, a significant (*p* < 0.01) difference in ER-α expression was detected between RBCs from early- and long-standing RA patients. Regarding ER-β expression, no significant differences have been found in RBCs from HD and RA patients (Figure 1C). The different expression of ER-α and ER-β also emerged by analyzing the typical flow cytometric profiles of the receptors content in RBCs from a representative HD, a representative early and a representative long-standing RA patient shown in Figures 1B,D. By using static cytometry, we also found that both ER-α and ER-β were mainly localized in the RBC membrane of early- and long-standing RA patients respect to HD. In addition, it is confirmed that, while the ER-β receptor does not show amount differences between early- and long-standing RA, the ER-α receptor is more expressed in the early phase. The micrographs show the distribution of ERs in the RBCs from a representative HD, a representative early-standing RA patient and a representative long-standing RA patient (Figure 2).

### Correlation between disease activity, oxidative stress, inflammation, and ER-α expression

DAS-28 score did not correlate with plasma TAC (R = - 0.71; *p* = 0.439). This result was expected because the high systemic pro-oxidant status, characterizing RA disease and its positive correlation with DAS-28 score (Quiñonez-Flores et al., 2016), inexorably leads to the depletion of the antioxidant systems (Da Fonseca et al., 2019). No correlation was found between p-ERK_1/2_ and DAS-28 and p-ERK_1/2_ and RF (results not shown). Surprisingly, a correlation was found between DAS-28 score and ER-α content (R = 0.22). These results suggested that the expression of ER-α on the RBCs membrane of RA patients might be linked to the exogenous inflammation and oxidative stress conditions. In addition, the binding of the hormone to the estrogen receptor present on RBCs membrane could activate the intracellular signaling, modulate the intracellular redox status (Di Franco et al., 2015; Vona et al., 2019), allowing RBCs to become in turn source of reactive oxidizing species.

### Correlations between the ER-α expression and the parameters linked to inflammation and oxidative stress

To evaluate a likely association between the ER expression and the parameters linked to the inflammation and oxidative stress, we correlated the ER-α expression with the 1) biomarkers of the disease acute-phase, 2) biomarkers of oxidative stress, and 3) expression of the phosphorylated form of ERK_1/2_ (p-ERK_1/2_), taken as a marker of the kinase-linked intracellular signaling activation (Table 2). We found that ER-α expression in RBCs from RA patients did not correlate with plasma TAC and correlated with 1) inflammatory biomarkers, i.e., ESR and CRP, 2) oxidative stress-related biomarkers, i.e., ROS/RNS and 3-nitrotyrosine (3-NT), and 3) p-ERK_1/2_ expression (Table 2).

**TABLE 2:** Correlation of ER-α with various selected variables.

Variables	R	*p* Values
DAS-28	0.22	0.350
TAC	−0.050	0.884
ESR	0.116	0.719
CRP	0.280	0.420
ROS/RNS	0.354	0.258
3-NT	0.545	0.066
p-ERK ½	0.550	0.124

DAS-28, disease activity score; TAC, total antioxidant capacity of plasma; ESR, erythrocyte sedimentation rate; CRP, C reactive protein; ROS, reactive oxygen species; RNS, reactive nitrogen species; 3-NT, 3-nitrotyrosine; p-ERK_1/2_, phosphorylated extracellular signal-regulated kinase. Student’s t-test was used for the statistical analysis of the collected data. Correlations were evaluated by using Pearson correlation (R: correlation coefficient).

### Patient treatment and follow up

As established by the therapeutic treatment for RA, early and long-standing RA patients were treated with the DMARDs methotrexate and anti-TNF-α, respectively, and monitored after 6 months long survey, i.e., at follow-up (F.U.). As expected, we found that, in comparison with recruitment (T_0_), 6 months after treatment, the disease activity was significantly reduced in methotrexate- (DAS-28: 3.2 ± 1.3) and anti-TNF-α-treated (DAS-28: 2.9 ± 05) in both early- and long-standing patients, respectively. In addition to DAS -28 we found that other clinical parameters such as RF, ESR and CRP improved 6 months after therapy (Table 3). Moreover, some erythroid parameters including RBCs number, hemoglobin (Hb), mean corpuscular volume (MCV), hematocrit and reticulocytes were investigated. For all erythroid parameters analyzed, no significant differences were detected between early-and long-standing RA patients. Both at T_0_ and at F.U. the values of these parameters were within the reference range (Table 4).

**TABLE 3:** Clinical characteristics of RA patients 6 months after therapy.

Variables	Early-standing disease (n = 19)	Long-standing disease (n = 13)	*p* Values
DAS 28 score	3.0 ± 2	3.0 ± 0.03	0.398
RF (IU/ml)	Median 50 (range 0.5–0.75)	Median 12 (range 0–18)	0.078
ESR (mm/h)	Median 20 (range 4—39)	Median 14 (range 3—25)	0.082
CRP (mg/L)	Median 8.23 (range 0.5—10)	Median 1.57 (range 0.1—7)	0.124

RA, rheumatoid arthritis; DAS, 28 score, disease activity score; RF, rheumatoid factor; ESR, erythrocyte sedimentation rate; CRP, C reactive protein.

**TABLE 4:** Erythroid parameters of RA patients.

Parameters	RA-patients T_0_	RA-patients F.U.	Reference values	*p* Values
RBCs (x106/ml)	4.43 ± 0.9	4.48 ± 0.7	4–5.5	0.75
Hb (g/dl)	12.87 ± 0.5	13 ± 0.2	12–16	0.85
MCV (fL)	86 ± 4	85 ± 7	80–96	0.93
Hematocrit (%)	39 ± 3	37 ± 4	37–46	0.32
Reticulocytes (109/L)	32 ± 0.9	35 ± 0.6	25–75	0.43

RBCs, red blood cells; Hb, hemoglobin; MCV, mean corpuscular volume.

**FIGURE 3:**
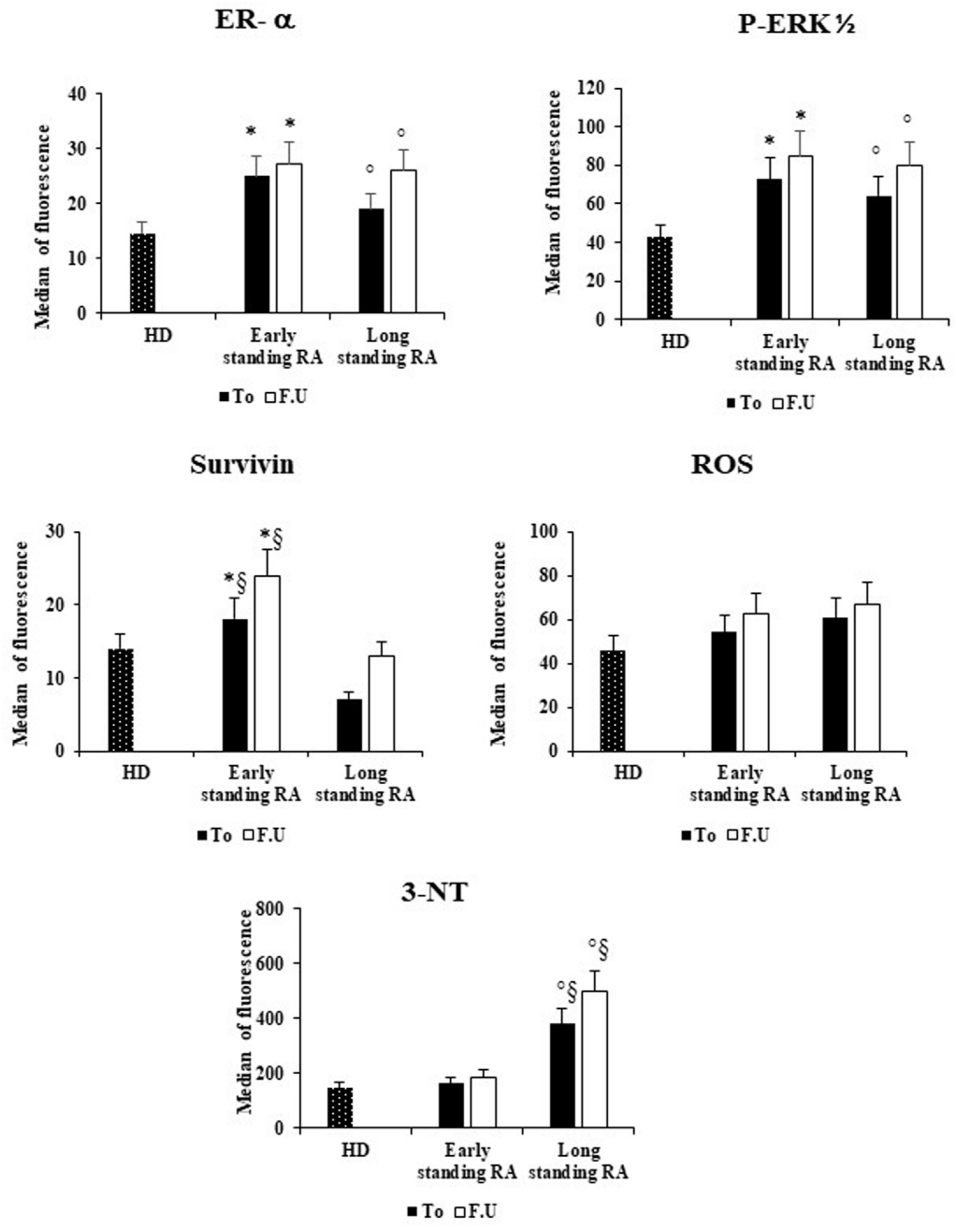
Effects of DMARS therapy on the ER-α expression and on the amounts of inflammatory and oxidative stress in RBCs of early- and long-standing RA patients. RBCs from healthy donors (HD), early- and long-standing RA patients were analyzed by flow cytometry analysis for the expression of ER-α, p-ERK_1/2_, and survivin, and for the content of ROS and 3-NT, before (T_0_) and after 6 months of DMARDs treatment (F.U.). *p < 0.05 early-standing RA (T_0_ and F.U.) vs*.* HD. ° *p* < 0.05 long-standing RA (T_0_ and F.U.) vs*.* HD. §*p* < 0.05 early-vs*.* long-standing RA (T_0_ and F.U.).

We then compared the data obtained in the RBCs from early-and long-standing RA patients before and after 6 months of therapy with the RBCs data from healthy donors (Figure 3). With respect to HD, we found that in RBCs from early -and long-standing RA patients, ER-α and p-ERK_1/2_ are significantly more expressed (*p* < 0.05) both at T_0_ and F.U. Moreover, with respect to RBCs from HD, survivin expression is significantly higher (*p* < 0.05) only in RBCs from early standing patients both at T_0_ and F.U. (Figure 3). Conversely, with respect to RBCs from HD, 3-NT content is significantly (*p* < 0.05) higher in RBCs from long standing patients both at T_0_ and F.U. Significant differences (*p* < 0.05) between early -and long-standing RA patients (both T_0_ and F.U.) were found only for survivin and 3-NT (Figure 3). Survivin is a survival molecule that, in a preliminary study, we found increased in RBCs from RA patients with respect to HD (Di Franco et al., 2015). For all variables analyzed there are no significant differences between RBCs from RA patients both at T_0_ and F.U. (results not shown). The pharmacological treatment with DMARDs increased not significantly the expression of ER-α and p-ERK_1/2_, as well as the levels of survivin and 3-NT in both group of RA patients (Figure 3).

## Discussion

Estrogens are believed to modulate several key features of the immune system, by regulating intracellular signaling linked to inflammation and oxidative stress, antigen specific responses, as well as cytokine production (Alpizar-Rodrıguez et al., 2017; Chakraborty et al., 2022). The estrogens effects depend on both their concentration and distribution of ER-expressing immune cells (Chakraborty et al., 2022). The same expression of ERs can be modulated by estrogen, as the case of ER-α, but not ER-β, and increased after the treatment of human fibroblast-like synovial cells with 17β-estradiol (Mitani et al., 2005). CD4^+^ T cells have been found to express almost high levels of ER-α in comparison to ER-β. The opposite occurs in B cells, while both CD8^+^ T cells and monocytes express low, but comparable levels of both ERs (Phiel et al., 2005). In synovial cells, sex hormones and their receptors have been previously investigated and both ER-α and ER-β have been identified in different types of synovial cells from RA patients with their expression being up-regulate by some inflammatory factors, such as IL-6 and IL-8 (Capellino et al., 2007).

In this work we found that both ER-α and ER-β were expressed in RBCs from RA patients, but only ER-α content was slightly increased in early diseased RA patients with respect to HD and long-standing RA patients. Similarly, to what detected in RBCs from HD, in this and in our previous work (Vona et al., 2019), the immunofluorescence study showed that in RBCs from RA patients, ER-α was mainly localized into the plasma membrane and its content was slightly higher in early-with respect to long-standing RA patients and HD. It is conceivable that in RA patients the localization of ER-α in the plasma membrane follows an estrogen-driven migration of the receptor from the cytosol in mature RBCs. Indeed, ER-α in healthy RBCs is mainly localized in the cytoplasm and it can be recruited to the membrane after activation by 17β estradiol or estrogen agonist (Vona et al., 2019). The membrane-bound ER-α has been reported to play a critical role in RBC homeostasis by stimulating the intracellular signaling linked to the kinase pathways (Vona et al., 2019). The activation of this non-genomic pathway, occurring in cells deprived of the nucleus such as RBCs, involves several important kinases, including the MAPK such as ERK_1/2_, AKT, and P38 (Chambliss et al., 2005; Osborne and Schiff, 2005; Nakaya et al., 2007; Lu et al., 2016). ERK_1/2_ in particular regulates critical cellular activities, such as cell adhesion and cell survival (Roskoski, 2012; Di Franco et al., 2015; Zou et al., 2019), and the level of its phosphorylated form has been found higher in RBCs from healthy females with respect to healthy males (Vona et al., 2019). Although in normal RBCs, ERK_1/2_ has been reported to undergo a maturation-related loss, in RBCs from sickle cell disease patients the kinase activities are preserved and linked to the signaling necessary for the RBCs altered adhesive functions (Zennadi et al., 2012). Our results go in the same direction supporting a role of ERs-related, ERK_1/2_-linked activation signaling in the outcome and, possibly, the progression of RA disease.

In general, due to the heterogeneity of symptoms duration and presence of damage, the clinical stages of RA cannot be differentiated on a chronological basis (Chatzidionysiou and Fragoulis, 2019). However, the analysis of the clinical profile of the early-stage RA patients involved in this study (including DAS-28 score, RF and CRP) let us confident about the high disease activity linked to the inflammatory condition compared to long-standing patients. Importantly, we showed for the first time that in RA patients RBCs, ER-α expression correlated with DAS-28 score. The correlation between DAS-28 score and RBCs ER-α expression associated the disease activity to the estrogen receptor-mediated activation of a signaling pathway also linked to the occurrence of mild oxidative stress and to the alteration of redox state in diseased RBCs (Vona et al., 2019). This statement is supported by the finding that RBCs from RA patients were found to undergo to a mild alteration in intracellular redox state associated with activation of ERK_1/2_ -related signaling. Moreover, the positive association between the ER-α expression, ERK_1/2_ phosphorylation and ROS/RNS production in RBCs further supported the hypothesis of involvement of a kinase-linked intracellular signaling whose activation, downstream to estrogen receptor, could play a key role in the induction of the mild oxidative stress detected in RBCs from RA patients (Vona et al., 2019).

The membrane-associated ER-α has been reported to mediate the eNOS phosphorylation and the NO production through a mechanism involving the activation of several kinases, including c-Src, phosphatidylinositol 3-Kinase (PI3K) and Akt (Mohanty et al., 2014; Pradhan et al., 2019). It has been reported that in RBCs, the activation of the eNOS isoform and the NO production were triggered by the ERK_1/2_ phosphorylation dependent on ER-α stimulation (Vona et al., 2019). Notably, in RA patients the link between ER-α and NO pathway was further suggested by the positive correlation between ER-α expression and 3-NT amount in RBCs. The formation of 3-NT is considered the footprint of RNS formation, with particular regards to peroxynitrite, the product of the fast reaction between O_2_^•^ and NO, as well as by the NO-derived nitrating species. In autoimmune diseases, tyrosine nitrated proteins have been hypothesized to behave as neo-antigens boosting the formation of autoantibodies (Paredes et al., 2002). The formation of peroxynitrite inside RBCs from RA patients is conceivable as a result of the increased 1) NO synthesis by the eNOS stimulated by ER-α activation and 2) O_2_^•^ formed as a result of hemoglobin auto-oxidation and activation of the NADH oxidases following the RBCs continuous exposition to exogenous oxidative insult (Mahdi et al., 2021; Mohanty et al., 2014).

Interestingly, both DAS-28 score and ER-α expression in RBCs of RA patients did not correlate with plasma TAC. While the former result confirmed by data reported by other groups (Paredes et al., 2002; Pradhan et al., 2019; Salem and Zahran, 2021), the lack of correlation between ER-α expression in RBCs and plasma TAC is, at our knowledge, a new finding. In fact, our study shows that an increase of the disease severity and the ER-α expression correlate with the increase of both systemic and intra-erythrocyte oxidative stress. It is indeed expectable that the severity of RA disease could be associated to a decreased TAC. The increased systemic oxidative stress condition, such as that occurring in tissues of RA patients (Kundu et al., 2012; Veselinovic et al., 2014; Quiñonez-Flores et al., 2016), could *per se* favor a RBCs pro-oxidant leading to the alteration of intracellular redox state and to the decrease of the intracellular antioxidant defense, such as glutathione (Alisi et al., 2021). Similarly, the negative correlation between ER-α expression and TAC indirectly links the increase of oxidative stress and the RBCs activation mediated by ER-α pathway which could further contribute to the establishment and promotion of the systemic pro-oxidant status of RBCs confirming their role as risk factors for CVD (Vona et al., 2019).

Several studies have shown a correlation between the disease severity and the parameters linked to oxidative stress in RA patients, reinforcing the association between oxidative/nitrative stress and the disease. In particular, DAS-28 score of RA patients has been correlated with the levels of lipid peroxidation products (Kundu et al., 2012; Datta et al., 2014; Quiñonez-Flores et al., 2016), with the formation of oxidizing species such as O_2_^•^, H_2_O_2_, ^•^OH and NO (Kundu et al., 2012; Veselinovic et al., 2014; Quiñonez-Flores et al., 2016), as well as the MPO-derived hypochlorous acid [33]. In addition, DAS-28 score also correlated with protein oxidation (Quiñonez-Flores et al., 2016), as well as with the low molecular weight antioxidant system impairment, such as the decrease of the levels of vitamins C and E, glutathione, β-carotene, zinc, and selenium (Quiñonez-Flores et al., 2016). High molecular weight antioxidants showed contrasting correlation with the disease activity being increased, decreased and even unaltered levels, such as in the case of superoxide dismutase, catalase, glutathione peroxidase, glutathione reductase and glutathione-S-transferase (Mateen et al., 2016). The correlation between RBC ER-α expression and oxidative stress biomarkers, as well as the lack of correlation with TAC, support a role of the estrogen receptor-linked pathway in the activation of RBCs of RA patients.

Regarding the different clinical stages of RA, our results indicate that RBCs from early-standing RA patients differ significantly (*p* < 0.05) from RBCs from long-standing RA patients in terms of survivin expression and 3-NT content. Survivin is an apoptotic pathway inhibitor and its increased expression in RBCs from patients with early-standing RA could indicate that, similarly to what has been reported for fibroblast-like lymphocytes and synoviocytes in the joints of RA patients (Zafari et al., 2019), RBCs from RA at this stage of the disease also survive for a long time as a consequence of the impaired apoptosis. Moreover, survivin up-regulation is induced by the p-ERK_1/2,_ that results, although not significantly, less expressed in long-standing RA patients. 3-NT is considered the footprint of RNS formation, with particular regards to peroxynitrite, the product of the fast reaction between O_2_^•^ and NO, as well as by the NO-derived nitrating species. Based on, we can hypothesize that in RBCs from long-standing RA patients’ reduction of p-ERK_1/2_ expression could lead to a decreased expression of survivin and consequently at an increase in RNS content. It is interesting to note that, under condition of oxidative stress, RBCs can activate a crosstalk between the antioxidant systems and activated kinase aimed to maintain cell homeostasis and survival. Is this the case of the oxidative stress-induced activation of peroxiredoxin-2 (Prx2), the third most abundant cytoplasmic protein in RBCs involved in the transduction of hydrogen peroxide signaling. Prx2 can be phosphorylated and activated by Syk, a Src family-related Tyr kinase involved in the redox signaling pathways (Mattè et al., 2021). On its hand, the chaperone function of Prx2 allows the enzyme to be involved in the activation of intracellular signaling depending on the cell type or stimuli (Latimer and Veal, 2016). Interestingly, ERK_1/2_ has been reported to induce *in vitro* the phosphorylation and activation of the isoform six of Prx2 (Elko et al., 2019). The likely crosstalk between ERK_1/2_ and Prx2 could contribute explain the increased survival of RBCs of early-standing RA patients and will be worth addressing in future investigations.

Interestingly, the treatment with DMARDs, if on the one hand improved the clinical condition measured by the reduction of DAS-28 score, on the other hand increased the oxidative stress-related biomarkers and the expression of survival-related proteins (Di Franco et al., 2015; Quiñonez-Flores et al., 2016; Da Fonseca et al., 2019). In our work, the treatment of RA patients with the DMARDs methotrexate and anti-TNF-α prescribed to early- and long-standing RA patients, respectively, increased RBC ER-α expression, p-ERK_1/2_ content, oxidative stress related parameters as well as the content of survivin, an inhibitor of the apoptotic pathway. It has been reported that the pro-oxidant effects induced by methotrexate are linked to reactive oxidizing species generation, antioxidant enzyme activity inhibition and decrease of low-molecular weight antioxidants (García-Sánchez et al., 2020). Moreover, differences in the clinical effectiveness of the therapy with anti- TNF-α has been found in RA patients, with particular regard to non-responder patients in which the circulating inflammatory profile and the oxidative stress biomarkers remained significantly elevated after 6 months of therapy (Luque-Tévar et al., 2021).

Although the DAS-28 score was decreased and indicated an improvement in the patient’s clinical condition, confirming the beneficial effects of DMARDs treatment, the increase in oxidative stress parameters linked to ER-α expression suggests a greater tendency of RBCs of RA patients in the pro-oxidant state associated with the expression of survival molecules, such as survivin. This molecule has been shown to play a critical role in RA conferring high survival to immune cells through apoptosis inhibition (Zafari et al., 2019). In addition, survivin has been found increased in RBCs from RA patients (Vona et al., 2019), as well as in RA synovial tissues and correlated with DAS-28 score (Ma et al., 2021). Its increased expression could indicate that, similarly to what reported for lymphocytes and fibroblast-like synoviocytes in joints of RA patients (Zafari et al., 2019), RA RBCs also survive for a long time as a consequence of compromised apoptosis. Since our results showed that the activation of ERK_1/2_ is linked to the expression of ER-α in RBCs from RA patients, the kinase phosphorylation could induce the upregulation of survivin increasing the risk of premature cardiovascular-related complications, such as heart failure, which characterizes RA disease (Kaplan, 2010; Zennadi et al., 2012). The phosphorylated form of ERK_1/2_ has been indeed reported to induce the up regulation of survivin, which, in turn, is recognized to favor the RBC aggregation and adhesion of other blood cells to the endothelium (Zennadi et al., 2012; Vona et al., 2019).

Considering the role of inflammation and oxidative stress in the development of RA and related CVDs, it is reasonable to hypothesize the use of compounds capable of counteract the formation of reactive oxidants linked to systemic inflammation. The potential pro-oxidant role of altered RBCs in RA could be weakened using antioxidants able to protect these cells from inflammation-related extracellular insults and deactivate subsequent intracellular dysfunction. With this regard, polyphenols have been shown to prevent a potential pro-oxidant activity of RBCs, as emerged by studies performed *in vitro* in the few recent years (Tedesco et al., 2016; Revin et al., 2019; Fernandes et al., 2020; Checkouri et al., 2020; Remigante et al., 2022). The *in vivo* administration of polyphenolic antioxidants in RA patients showed potential promising effects in alleviating the disease activity and to reduce blood biomarkers of inflammation and oxidative stress (Ghavipour et al., 2017). A more modest protective effect has been measured by using N-acetylcysteine, administered *in vivo* in a randomized double-blind placebo-controlled trial involving RA patients, whose weak antioxidant activity led the authors to suggest the compound as suitable as an adjunctive therapy (Esalatmanesh et al., 2022).

In conclusion, it is clear from our results that, despite the difficulty to clearly differentiate the clinical stages of RA on a chronological basis, RBCs of RA patients could behave as pathophysiological mediators and represent a risk factor in the occurrence of CVD linked to the disease. The dysfunctional RBCs could favor altered interactions with other cell types, transferring the signaling to adjacent cells, such as platelets, endothelial cells, and phagocytes, and contributing to the occurrence of pathophysiological conditions characterizing the RA disease. A better understanding of the role of ERs, in particular of ER-α, in the activation of RBCs signaling could then open new paths in therapeutic strategies based also on the use of ER selective agonists.

## Data Availability

The original contributions presented in the study are included in the article/supplementary material, further inquiries can be directed to the corresponding author.
